# Melæna massif révélant un syndrome de Peutz-Jeghers vu au CHU-JRA Madagascar: à propos d'un cas

**DOI:** 10.11604/pamj.2016.23.78.8862

**Published:** 2016-03-10

**Authors:** Andrianimaro Florelia Martinetti, Rasoanaivo Mamy Andriantsoa, Rajaonera Tovohery Andriambelo, Rakotoarison Ratsaraharimanana Cathérine Nicole, Raveloson Nasolotsiry Enintsoa

**Affiliations:** 1 Service Bloc opératoire-réanimation, CHRD Moramanga, Madagascar; 2 Service Réanimation Chirurgicale, Hôpital Joseph Ravoahangy Andrianavalona, CHU Antananarivo, Madagascar; 3 Accueil-Triage-Urgences-Réanimation, Hôpital Joseph Ravoahangy Andrianavalona, CHU Antananarivo, Madagascar; 4 Accueil-Triage-Urgences-Réanimation Médicale, Hôpital Joseph Raseta de Befelatanana, CHU Antananarivo Madagascar

**Keywords:** Choc, endoscopie, melaena, Peutz-Jeghers, polype, Shock, endoscopy, melaena, polyp, Peutz-Jeghers syndrome

## Abstract

Le syndrome de Peutz-Jeghers (SPJ) est caractérisé par l'association d'une polypose digestive hamartomateuse et d'une lentiginose cutanéo-muqueuse. Les malades sont exposés à des complications mécaniques et hémorragiques. Il s'agit d'un syndrome de prédisposition au cancer. Notre étude a pour objectif de rappeler les diagnostiques d'un syndrome de Peutz-Jeghers, de connaitre les complications ainsi que les progrès thérapeutiques dans la prise en charge. Nous avons rapporté le cas d'un homme de 32 ans présentant un melaena massif. Il a été hospitalisé en service de réanimation chirurgicale pour état de choc hypovolémique difficile à contrôler. Il a nécessité une intervention chirurgicale pour arrêter l'hémorragie. Nous avons trouvé un polype hamartomateux dans le grêle qui a causé le saignement. Le diagnostic d'un Syndrome de Peutz-Jeghers a été posé devant la notion de lentiginose labiale pendant l'enfance. Lors de l'exploration clinique et paraclinique, il ne présente pas encore de cancer. A Madagascar, cette pathologie est encore mal connue. Dans la littérature, le syndrome de Peutz-Jeghers peut être révélé cliniquement ou au stade de complication comme l'hémorragie, l'invagination ou l'occlusion intestinale. Dans notre cas, la maladie est compliquée d'une hémorragie digestive avec état de choc hypovolémique. La polypectomie endoscopique par entéroscopie à double ballonnet permet de diminuer le recours à la chirurgie grêlique d'urgence. Le syndrome de Peutz-Jeghers est une affection rare. Mais il est important aux cliniciens de le connaitre et de penser à sa possibilité en cas d'hémorragie digestive.

## Abstract (en)

Peutz-Jeghers syndrome (SPJ) is characterized by intestinal hamartomatous polyps in association with mucocutaneous lentiginosis. Patients are exposed to mechanical and bleeding complications. It is a cancer predisposition syndrome. Our study highlights the diagnostic criteria for Peutz-Jeghers syndrome (SPJ), the complications and the therapeutic progresses in patient care. We report the case of a 32-year-old male presenting with a massive melaena. It was hospitalized in the surgical intensive care unit with hypovolemic shock difficult to control. This required surgical intervention to stop bleeding. We found a hamartomatous polyps in the small intestine which caused bleeding. Peutz Jeghers Syndrome was diagnosed on the basis of labial lentigines during childhood. Clinical and paraclinical explorations did not reveal the presence of cancer. In Madagascar, this disease is still poorly understood. In the literature, the diagnosis of Peutz Jeghers syndrome is based on clinical findings or on the presence of complications such as haemorrhage, invagination or bowel obstruction. In our case, the disease was complicated by gastrointestinal bleeding with hypovolemic shock. Endoscopic polypectomy using double-balloon enteroscopy can reduce emergency small bowel surgery. Peutz-Jeghers syndrome is a rare disease. Despite this, it is important for clinicians to know it and to take it into consideration in case of gastrointestinal bleeding.

## Introduction

Le syndrome de Peutz-Jeghers (SPJ) est caractérisé par l'association d'une polypose digestive, et une lentiginose cutanéo-muqueuse à prédominance péri-orificielle. C´est une affection rare, héréditaire, à transmission autosomique dominante. Il est parfois associé à des tumeurs malignes, digestives ou extra-digestives. La polypose intestinale en constitue la composante essentielle [1]. Nous rapportons le cas d'un homme de 32 ans entré dans le service de Réanimation Chirurgicale du Centre Hospitalier Universitaire Joseph Ravoahangy Andrianavalona Antananarivo (CHU-JRA) pour un melaena massif nécessitant des transfusions sanguines et une intervention chirurgicale en urgence. Le syndrome de Peutz-Jeghers a été posé devant le polype hamartomateux présent dans l'intestin grêle et la notion de lentiginose sur ses lèvres pendant son enfance. Le but de ce travail est de rappeler les diagnostiques d'un syndrome de Peutz-Jeghers, de connaitre les complications ainsi que les progrès thérapeutiques dans la prise en charge de ce syndrome à la lumière d'une revue de la littérature. Nous soulignerons également l'intérêt d'une surveillance périodique pour dépister la survenue de cancer.

## Patient et observation

Il s'agit de monsieur M. âgé de 32 ans, marié, commerçant, pesant 49 Kg et mesure 1,60m. Il est entré dans le service de Réanimation chirurgicale Hôpital joseph Ravoahangy Andrianavalona pour une hémorragie digestive à type de melaena. Depuis une semaine avant son admission, le malade éprouve des malaises digestifs sous forme de coliques abdominales localisées surtout au niveau de la partie inferieure de l'abdomen. La douleur est surtout maximale après la prise alimentaire. Elle s'aggrave et devient diffuse. Le patient consulte un médecin qui lui prescrit un anti spasmodique. Mais, malgré ce traitement il n'y a pas d'amélioration, son état général s'altère, il perd l'appétit et ses selles deviennent rouges noirâtres, d'où son hospitalisation dans le service de Réanimation Chirurgicale HJRA. Une échographie abdominale est effectuée et ne révèle aucune anomalie. Lors d'une enquête étiologique, il a présenté des douleurs abdominales à répétition depuis l’âge de 15 ans et il a mentionné une notion des petites taches noires au niveau de ses lèvres vers l’âge de 4 ans qui disparaissent progressivement jusqu’à l’âge de 30 ans. Depuis un an, il a présenté une épigastralgie à répétition mais il n'a reçu aucun traitement adapté. Il n'a aucun antécédent chirurgical. C'est un sujet alcoolique, mais non tabagique. Ses parents sont encore en vie et n'ont aucun antécédent particulier. Il est l'ainé d'une fratrie de trois dont les deux autres filles sont en bonne santé apparente. Il est père d'une fille de 4 ans et d'un garçon de 2 ans qui sont tous les deux en bonne santé; et d'après l'interrogatoire aucune tache n'est trouvée sur leurs corps. À l´admission, l´examen clinique a trouvé un patient conscient avec un état général altéré, et des conjonctives très pâles. Il avait une pression artérielle basse (90>50mmHg); et une tachycardie à 106 battements par minute (bpm).

L'examen physique a montré un abdomen de volume normal, souple mais douloureux à la palpation de l'hypogastre. L'examen des autres appareils a été normal. Arrivé en réanimation, nous avons mis en condition le malade. En plus d'une voie veineuse déjà posée au service des urgences, nous avons posé une deuxième voie veineuse 18G au niveau de son bras. Nous avons pratiqué un prélèvement sanguin pour analyse biologique et pour le groupage sanguin. Nous avons procédé au remplissage vasculaire par des macromolécules (Hydroxyéthylamidon: Héstar^®^) 500ml et cristalloïdes (sérum salé isotonique 0,9% et Ringer Lactate) 1000mL par 24 heures en attendant les résultats biologiques. Le patient a été gardé à jeun. L'apport calorique a été assuré par du sérum glucosé hypertonique 10% (1000mL par 24 heures). Il a reçu un inhibiteur de pompe à proton (Omeprazole) 40mg par jour, et des électrolytes, KCl 3g/j.

Une demande de fibroscopie digestive haute d'urgence a été faite. Un lavement évacuateur a été faite chaque jour pour évaluer les caractères de saignement et pour évacuer les selles. Les surveillances de l’état hémodynamique ont été rapprochées: Pression Artérielle (PA), Fréquence Cardiaque (FC), diurèse: toutes les heures au minimum. L'apport hydrique était modifié en fonction du bilan hydrique. Au deuxième jour: L'hypotension artérielle a persisté (PA: 90>50mmHg; FC: 105bpm) Le lavement évacuateur a été noirâtre. La numération de la formule sanguine (NFS) a révélé: une anémie microcytaire normochrome assez sévère; Hémoglobine (Hb): 78g/dL; VGM: 84µ; CCMH: 334 g/L; un taux de plaquettes normal (157 10^9^/L). L'hémostase secondaire a été normale (TP:89,05%; TCA: 28,8s/29,3s) Les résultats de la glycémie, urée, créatininémie, ionogramme sanguin sont normaux. En plus des mesures précédentes nous avons commencé la transfusion sanguine par deux culots globulaires (CG) iso-groupe iso-rhésus et une poche de plasma frais congelé (PFC). Au troisième jour: malgré notre réanimation, la pression artérielle reste toujours basse (PA: 90/50mmHg; FC: 96bpm). La fibroscopie oeso-gastroduodénale (FOGD) a montré une muqueuse œsophagienne normale, le cardia a été normal. Par contre, l'exploration de l'estomac a montré un lac muqueux hémorragique qui a gêné l'examen. Le contrôle de la NFS a montré encore une anémie normocytaire normochrome assez sévère (Hb: 74 g/L) et une thrombopénie modérée (Plaquettes: 109 10^9^/L) Nous avons continué la transfusion sanguine par des culots globulaires et du PFC. Au sixième jour: La pression artérielle reste toujours basse, le lavement évacuateur devient rouge. L'anémie a persisté (Hb: 73g/L) accompagnée d'une thrombopénie (plaquette: 76.10^9^/L). La reprise de la FOGD a montré un lac muqueux sanguinolent d'abondance normale dans l'estomac. Au niveau de la face postérieure du fundus ils ont trouvé un ulcère plus ou moins vaste à fond blanchâtre avec du caillot adhérent à la périphérie. Le diagnostic d'une hémorragie digestive haute par un ulcère gastrique a été posé. Nous avons démarré la trithérapie par: Deux antibiotiques: Amoxicilline 1g trois fois par jour Metronidazole 500mg trois fois par jour, Un inhibiteur de pompe à proton: Oméprazole (Lomac^®^) 40mg par jour. Au septième jour: Le patient est tombé brutalement dans un état de choc hémorragique sévère, avec une perte de connaissance, sa pression artérielle a chuté (PA:70>50mmHg avec FC: 110 bpm), ses selles étaient rouges noirâtres. La NFS d'urgence a montré une anémie normocytaire normochrome assez sévère. (Hb: 69g/L; VGM: 86µ3; CCMH: 335g/L). Nous avons mis le patient en tête basse (position de Trendelenburg). Nous avons apporté de l'oxygène par des lunettes nasales à 6L/min avec un remplissage vasculaire par transfusion de culot globulaire iso groupe iso-rhésus. Au huitième jour: Le remplissage vasculaire et la transfusion sanguine n'arrivent pas à rétablir l’état hémodynamique du patient. Nous avons demandé l'avis du chirurgien de garde qui a posé l'indication d'une intervention chirurgicale d'urgence pour faire l'hémostase. Nous avons préparé le malade pour l'intervention. Nous avons mis en place une voie veineuse centrale sous-clavière droite16G. L'hypovolémie a été corrigée par une perfusion de colloïdes (Héstar^®^ 500 ml) et par une transfusion de trois poches de sang total iso groupes iso-rhésus. Le patient a été toujours gardé à jeun. Après quelques heures de préparation, il a été prêt pour l'intervention. Il a eu une PA à 110> 60 mmHg avec une FC à 90 bpm.

L'intervention consistait à une laparotomie médiane sous ombilicale. Après une gastrotomie, on n'a trouvé que du liquide gastrique (absence de sang). La recherche de la lésion a objectivé un ulcère déjà cicatrisé sans réaction inflammatoire, donc fermeture de la gastrotomie. Une vérification sur l'intestin grêle a retrouvé une petite tumeur polypoïde sessile; 1 cm de diamètre qui se trouve à 30cm de l'iléon terminal. On a procédé à l'ablation de la tumeur, fermeture de l'intestin grêle et fermeture plan par plan de la paroi. La pièce opératoire a été envoyée pour examen anatomo-cyto-pathologique. Aucun incident n'est trouvé pendant l'intervention, son état hémodynamique reste stable.

**Figure 1:**
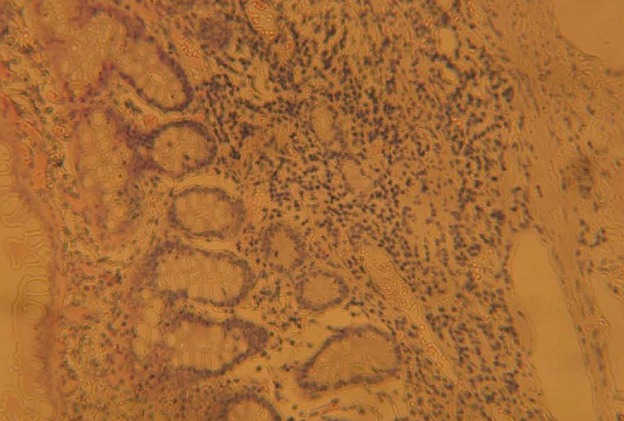
Coupe histologique de la pièce opératoire montrant des structures glandulaires hamartomateuses accompagnées de faisceaux de fibres musculaires lisses

Les suites opératoires étaient simples, elles étaient marquées par une amélioration; stabilisation de l’état hémodynamique; une reprise du transit au deuxième jour postopératoire. Le lavement évacuateur est devenu clair au bout de trois jours. Le traitement post opératoire comportait à une rééquilibration hydro-électrolytique guidée par le bilan hydrique et les résultats des analyses biologiques, une alimentation parentérale par de l'Oliclinomel^®^ 1L par jour, une antibiothérapie; un inhibiteur de pompe à proton; un antalgique. L'alimentation orale a été débutée à partir du 5^ème^ jour postopératoire par des aliments mous. Le résultat de l'examen d'anatomie pathologique de la pièce opératoire a montré qu'il ne s'agit pas d'une tumeur maligne. La muqueuse est d'aspect normal. Au niveau de la sous-muqueuse ils ont observé des structures glandulaires hamartomateuses accompagnées de faisceaux de fibres musculaires lisses et de gros vaisseaux dilatés et congestifs. L'aspect de la lésion évoque un polype hamartomateux (Figure 1).

Le diagnostic d'un syndrome de Peutz-Jeghers a été posé devant l'association de ce polype hamartomateux et la notion de lentiginose sur ses lèvres dans son enfance. Le patient a bénéficié par la suite d'un examen clinique minutieux, d´une échographie abdominale et prostatique, à la recherche de cancer. Les examens se sont révélés sans particularités. On lui a bien expliqué le syndrome de Peutz-Jeghers, le risque de récidive de complication, le risque de cancer à de très jeune âge, et les surveillances périodiques qu'il doit faire. Le patient est sorti de l'hôpital au 10^ème^ jour postopératoire. Depuis, on n'a plus des nouvelles de lui.

## Discussion

Le syndrome de Peutz-Jeghers est une maladie rare, mais non exceptionnelle. La fréquence mondiale est estimée à 1/200 000 naissances mais elle diffère entre chaque étude; aux Etats-Unis il est estimé à 1 /60 000 à 1/300 000 naissances. C'est une pathologie héréditaire à transmission autosomique dominante. Les deux genres sont atteints avec une égale répartition. Il peut se produire dans tout groupe racial ou ethnique [2, 3].

A Madagascar, le syndrome de Peutz- Jeghers est encore mal connu. Notre cas serait le premier rapporté dans notre pays. Cela peut être due à l'insuffisance des examens d'anatomie pathologique pour faire son diagnostique, ou due à une méconnaissance de ce syndrome. C'est pourquoi le diagnostic n'est posé qu'au stade tardif de la maladie. Notre patient présentait une hémorragie digestive associée à un état de choc hypovolémique difficile à contrôler. L’évolution pourrait être fatale car il y a un risque de coagulation intra-vasculaire disséminée et collapsus cardiovasculaire. Le syndrome de Peutz-Jeghers est caractérisé par l'association de polype hamartomateux gastro-intestinal et d'une hyperpigmentation cutanéo-muqueuse. La polypose se développe sur l´ensemble du tractus digestif, à l´exception de la bouche. Histologiquement, les polypes sont de nature hamartomateuse correspondant à des polypes d´architecture tubulovilleuse sans dysplasie. L´axe des villosités a pour caractéristique de comporter des faisceaux de fibres musculaires lisses ce qui les différencie des adénomes. Les tubes sont revêtus de cellules cylindriques de haute taille, tantôt entérocytaires, tantôt caliciformes, à mucosecrétion augmentée. Généralement, ces polypes n'ont pas de potentiel malin [3].

Dans notre cas le polype s'est manifesté à l’âge de 32 ans par une hémorragie digestive. Le polype s'est trouvé dans l'intestin grêle; et vu lors d'une exploration chirurgicale. L'examen d'anatomie pathologique confirme qu'il s'agissait d'un polype hamartomateux (Figure 1). La lentiginose périorificielle, ou hyperpigmentation cutanéo-muqueuse est rarement présente à la naissance. Dans la littérature elle apparait avant la cinquième année de vie puis décroît progressivement à l´âge adulte pour parfois disparaître complètement, elle est maximale à la puberté. Elle résulte de dépôts cutanés et/ou muqueux de mélanine réalisant des macules hyper pigmentées brunes foncées ou noires environ 1 à 5 mm de diamètre. Elle est quasi constante mais non pathognomonique, puisqu´un aspect similaire, mais souvent moins spectaculaire peut être observé chez certains sujets normaux; elle intéresse essentiellement les pourtours de l´orifice buccal (94%), des yeux et des narines (66%), les mains et les pieds (62%); la muqueuse péri-anale est beaucoup plus rarement atteinte [4]. Elle précède habituellement la survenue des polypes. Chez notre patient, la lentiginose périorificielle a intéressé ses lèvres. Elle a été présente dans son enfance et a été disparue avant son âge adulte. Le diagnostic de syndrome de Peutz-Jeghers a été posé devant l'association de lentiginose péri-orificielle et le polype hamartomateux du grêle ainsi que son âge. Le syndrome de Peutz-Jeghers est caractérisé par son risque élevé de cancer, aussi bien digestif qu'extra-digestif. D'après Lim et al, dans une étude de 240 patients atteints de SPJ, le risque de survenue de cancer est de 1% avant l’âge de 20 ans; 19% avant l’âge de 40 ans; 63% avant l’âge de 60 ans et 81% avant l’âge de 70 ans [5]. Dans ses études sur 419 malades de SPJ, Hearle et al ont trouvé des résultats sur la fréquence de cancer qui a été semblable à ce que Lim a trouvé. Les cancers les plus fréquents dans cette analyse ont été les cancers digestifs. Pour les cancers extra-digestifs, le cancer du sein a été le plus fréquent [6]. Lim et al ont rapporté que 8% de femmes avec le SPJ ont développé un cancer du sein avant l’âge de 40 ans; 32% avant l’âge de 60 ans [5]. Dans notre cas nous n'avons pas encore dépisté l'existence de cancer chez notre patient. Dans le syndrome de Peutz-Jeghers, la polypéctomie est recommandée pour tout polype de plus de 1 cm de diamètre en raison d'un risque de complication. L´hémorragie, l´invagination ou l´occlusion intestinale représentent une indication opératoire indiscutable. Il faut garder à l´esprit que les complications peuvent être récidivantes. Donc il faut éviter dans la mesure du possible les résections intestinales trop étendues pouvant conduire à terme à un grêle court. Actuellement deux techniques permettent de réséquer les polypes du SPJ: l'entéroscopie per-opératoire et l'entéroscopie à double ballonnet. Cette technique permet de diminuer le recours à la résection intestinale, et permet de reséquer tous les polypes présents dans le tube digestif [7].

Dans notre cas, l'indication chirurgicale a été posée devant le caractère actif du melaena et devant un état hémodynamique instable malgré notre remplissage vasculaire. Nous avons cru que c'est le vaste ulcère présent dans la face postérieure du fundus qui a saigné. Mais lors de l'exploration chirurgicale, nous avons constaté que ce n'est pas le cas. L'exploration de l'intestin grêle a montré une petite tumeur, l'exérèse a été faite par une méthode chirurgicale seulement. Nous n'avons pas pu effectuer l'entéroscopie per-opératoire faute de moyen. Nous ne sommes pas arrivés à explorer tout l'intestin. Actuellement, depuis l'avènement de l'entéroscopie à double ballonnet (EDB), un traitement moins agressif des polypes du grêle est possible. Dans le syndrome de Peutz-Jeghers, la polypéctomie endoscopique par EDB a permis de diminuer le recours à la chirurgie grêlique d'urgence [8]. Dans leurs études en 2007, May et al ont montré l'intérêt de l'entéroscopie à double ballonnet dans le traitement de polypes intestinaux. Ils ont réalisé 44 polypéctomies chez 19 patients. 36% seulement des polypes se trouvaient au niveau du jéjunum proximal (limite de progression de l'entéroscopie poussée) et 16% étaient au niveau de l'iléon terminal (limite de la coloscopie standard avec iléo scopie). De fait 50% des polypes réséqués ne pouvaient être atteints que par une EDB [9]. Bien que cette technique soit particulièrement prometteuse, il existe des limites à son application. Elle nécessite un opérateur expérimenté et l'examen est long. Les complications de la polypéctomie, et en particulier le risque de perforation, sont estimées entre 4 et 10%.

L´évolution du SPJ ainsi que la survie moyenne des patients sont difficiles à préciser. En général, ce syndrome est considéré comme une affection bénigne, compatible avec une survie prolongée sous une surveillance médicale à vie, mais cette évolution est menacée par la survenue d´une occlusion intestinale aiguë, et d'une hémorragie parfois récidivante, multipliant les interventions chirurgicales avec leurs risques propres et exposant aux troubles nutritionnels secondaires à un syndrome du grêle court [10]. Ainsi les patients atteints de maladie de Peutz-Jeghers méritent une surveillance particulière. Il faut dépister précocement la survenue de polype à l'aide d'un examen endoscopique périodique. Tout les deux à trois ans: une échographie abdomino-pelvienne et prostatique, une endoscopie digestive complète (haute et basse), un transit intestinal, un entéroscanner. Pour notre cas, nous avons programmé pour le patient une surveillance clinique tous les ans, une fibroscopie haute et basse tout les deux ans. Mais depuis sa sortie de l'hôpital nous n'avons plus de nouvelles de lui.

## Conclusion

Le syndrome de Peutz-Jeghers est une affection rare. C'est un syndrome à prédisposition au cancer. Notre étude a montré un polype hamartomateux de Peutz-Jeghers compliqué d'un melaena massif avec un état de choc hypovolémique qui a nécessité une intervention chirurgicale en urgence. La polypéctomie endoscopique a un très grand intérêt dans la prise en charge de ce syndrome pour éviter l'intervention chirurgicale répétitive. Un protocole de surveillance doit être établi à chaque patient atteint de SPJ pendant toute leur vie pour éviter les complications. Bien que la fréquence de ce SPJ soit basse, il est important aux cliniciens de le connaitre et de penser à sa possibilité en cas d´hémorragie digestive.
